# Efficacy and safety of high-flow nasal cannula versus continuous positive airway pressure and conventional oxygen therapies in pediatric acute respiratory failure: a systematic review and meta-analysis

**DOI:** 10.1186/s12887-026-07220-8

**Published:** 2026-07-01

**Authors:** Elmira Haji Esmaeil Memar, Rohola Shirzadi, Negin Sarhang Rahsepar, Sara Ashtari

**Affiliations:** 1 Pediatric Center of Excellence/Children’s Medical Center, Tehran University of Medical Sciences, Tehran, Iran; 2 Pediatric Respiratory and Sleep Medicine Research Center, Children’s Medical Center, Tehran University of Medical Sciences, No. 62, Dr. Mohammad Gharib Street, Tehran, 1419733151 Iran; 3 Research Institute for Gastroenterology and Liver Diseases, Shahid Beheshti University of Medical Sciences, Tehran, Iran

**Keywords:** Oxygen inhalation therapy, High-flow nasal cannula, Continuous positive airway pressure, Pediatric, Respiratory failure

## Abstract

**Background:**

Pediatric acute respiratory failure (ARF) is a significant clinical challenge, often requiring respiratory support. While Continuous Positive Airway Pressure (CPAP) and conventional oxygen therapy (COT) are standard treatments, High-Flow Nasal Cannula (HFNC) has emerged as a promising non-invasive alternative, though its benefits remain unclear. This study aimed to compare the efficacy and safety of HFNC versus CPAP and COT in treating pediatric ARF.

**Methods:**

A literature search was conducted in Embase, PubMed, Cochrane Library, Scopus, and Web of Science until June 15, 2026. Randomized controlled trials (RCTs) comparing HFNC with CPAP and COT in children with ARF secondary to acute severe asthma, bronchiolitis, or pneumonia were included. Primary outcomes were treatment failure and intubation rates; secondary outcomes included oxygen therapy duration, pediatric intensive care unit (PICU) length of stay (LOS), hospital LOS, and mortality. Quality assessment used the Cochrane Risk of Bias Tool, and a random-effects meta-analysis was performed to calculate pooled odds ratios (ORs) and mean differences (MDs).

**Results:**

A total of 22 RCTs involving 6,641 children were included in this analysis. Compared to COT, HFNC was associated with a significantly lower risk of treatment failure (OR: 0.509, 95% CI: 0.42–0.61). However, there were no significant differences in intubation rates (OR: 0.796, 95% CI: 0.39–1.63) or hospital mortality (OR: 0.654, 95% CI: 0.4–1.06) between the HFNC and COT groups. Compared to CPAP, HFNC showed increased risks for treatment failure (OR: 1.343, 95% CI: 0.75–2.4), intubation rates (OR: 1.044, 95% CI: 0.58–1.89), and hospital mortality (OR: 3.11, 95% CI: 0.96–10.1), although these increases did not reach statistical significance (*P* > 0.05). Additionally, there were no significant differences in the duration of oxygen therapy, pediatric PICU LOS, or hospital LOS between HFNC and either CPAP or COT.

**Conclusions:**

HFNC significantly reduces the risk of treatment failure compared to conventional oxygen therapy in children with ARF, though it does not differ significantly from CPAP regarding intubation rates or hospital mortality. Further research is necessary to clarify its relative efficacy and safety in comparison to CPAP.

**Clinical trial registration:**

CRD420250652913

**Supplementary Information:**

The online version contains supplementary material available at https://doi.org/10.1186/s12887-026-07220-8.

## Background

Pediatric acute respiratory failure (ARF) is a significant clinical challenge, defined as the inability to maintain adequate gas exchange, manifesting as hypoxemia, hypercapnia, or increased work of breathing, and contributing to approximately 30% of admissions in pediatric intensive care units (PICUs) [1, 2]. The management of pediatrics ARF, particularly in common conditions such as asthma, bronchiolitis, and pneumonia, requires a range of treatment modalities aimed at enhancing oxygenation and improving respiratory mechanics [3, 4]. Among these interventions, High-Flow Nasal Cannula (HFNC) therapy has garnered significant attention as a promising alternative to conventional oxygen therapy (COT) and non-invasive ventilation strategies, including nasal Continuous Positive Airway Pressure (CPAP) and Bi-level Positive Airway Pressure (BiPAP) [5, 6].

HFNC delivers a high flow of warmed and humidified oxygen through nasal cannulae, providing both supplemental oxygen and an aerosolized flow that may alleviate the work of breathing and enhance patient compliance [7, 8]. Its increasing use in pediatric populations can be attributed to its tolerability, safety, and ease of use compared to non-invasive ventilation (NIV) devices [9]. The straightforward application of HFNC has led to its widespread use in various clinical settings, including emergency departments and general pediatric wars [7–9]. Despite positive feedback from healthcare providers, the existing literature presents mixed findings [10, 11]; some studies support HFNC [12, 13], while others favor COT, CPAP, or other NIV devices [14, 15]. This variability, combined with the urgent need for evidence-based interventions, underscores the necessity for a focused meta-analysis of randomized controlled trials (RCTs).

Although several systematic reviews and meta-analyses have assessed the efficacy of HFNC compared to CPAP and COT, their findings remain controversial. Dafydd et al. [16], found no significant difference in treatment failure rates between CPAP and HFNC in children with bronchiolitis, while Wang et al. [17], and Buendía et al. [18], reported that CPAP was associated with a lower risk of treatment failure in children with acute lower respiratory infections and acute bronchiolitis, respectively. Moreover, recent clinical trials have been published that were not incorporated into earlier systematic reviews, revealing a critical gap in the current evidence base. Much of the existing literature has also predominantly focused on acute bronchiolitis, with limited research addressing other conditions related to ARF, particularly in cases involving acute asthma exacerbations, bronchiolitis, and pneumonia. Given these discrepancies and the urgent need for comprehensive, updated insights into the efficacy and safety of HFNC compared to CPAP and COT, it is essential to synthesize the available data to provide clear guidance for pediatric clinicians.

The objective of this systematic review and meta-analysis was to review and synthesize the existing literature on high-quality RCTs regarding the effects and safety of HFNC therapy in comparison to CPAP and COT in children diagnosed with ARF, particularly in cases involving acute asthma exacerbations, bronchiolitis, and pneumonia. As the landscape of pediatric respiratory care continues to evolve, this meta-analysis aimed to clarify the comparative effectiveness and safety of HFNC relative to alternative interventions. By aggregating data from these RCTs, we sought to provide valuable insights that would enhance management strategies for pediatric patients experiencing respiratory emergencies.

## Methods

### Design and registration

The study protocol was rigorously registered with PROSPERO, the International Prospective Register of Systematic Reviews, under the unique code (CRD420250652913). This systematic review strictly adhered to the guidelines set forth by the Preferred Reporting Items for Systematic Reviews and Meta-Analyses (PRISMA-P) 2020 framework [19].

### Search strategy

We conducted a comprehensive literature search across multiple databases, including Embase, PubMed, Cochrane Library, Scopus, and Web of Science, with the search concluding on June 15, 2026. Relevant studies were identified using a combination of appropriate keywords and Medical Subject Headings (MeSH) terms as follows: (“oxygen inhalation therapy” OR “high-flow nasal cannula*” OR “high flow cannula*” OR “high flow oxygen therapy” OR “high flow oxygen” OR “high flow therapy” OR “HFNC”) AND (“Continuous Positive Airway Pressure” OR “CPAP ventilat*” OR “airway pressure release ventilat*” OR “nasal continuous positive airway pressure” OR “nCPAP ventilat*” OR “CPAP” OR “nCPAP”) AND (“conventional oxygen therapy” OR “COT” OR “Standard oxygen therapy” OR “SOT” OR “Nasal Cannula” OR “Nasal Prong” OR “Nasal Catheter” OR “Oxygen Mask” OR “Mask”) AND (“Pediatrics” OR “children” OR “young patients” OR “teenagers” OR “adolescent”) AND (“Acute Respiratory Distress” OR “Acute Respiratory Failure” OR “ARF” OR “PARF”) AND (“Bronchiolitis” OR “Bronchiolitides”) AND (“Pneumonia” OR “Experimental Lung Inflammation” OR “Pulmonary Inflammation”) AND (“Asthma” OR “Bronchial Asthma”). An example of the search strategy used for PubMed, Scopus and Web of Science can be found in (Supplementary File 1, Table S1). In addition to published literature, we explored grey literature (i.e., unpublished studies) using the national grey literature collection (https://allcatsrgrey.org.uk). Preprint servers such as medRxiv (https://www.medrxiv.org) and PsyArXiv (https://psyarxiv.com) were also searched. Furthermore, we performed an incognito search in Google Scholar, reviewing the first 200 results for potential inclusion. The EThOS database (https://ethos.bl.uk) was consulted as well. Additionally, we manually reviewed the reference lists of potentially eligible papers to ensure that no relevant studies were overlooked. To maintain the relevance and applicability of our findings, we included only studies published in English that involved human subjects, without imposing any date restrictions.

### Eligibility criteria

The eligibility criteria for studies included in the analysis were determined using the PECOs strategy, which stands for Participants, Exposure, Comparison, Outcome, and Study Design [20]. (P) population: children aged 18 years or younger diagnosed with Acute Respiratory Failure (ARF) or Acute Respiratory Distress requiring supplemental oxygen or non-invasive respiratory support. For the purpose of this review, ARF is defined as the inability to maintain adequate gas exchange, manifesting as hypoxemia, hypercapnia, or increased work of breathing, secondary to acute lower respiratory tract infections (bronchiolitis, pneumonia) or acute severe asthma exacerbations [1, 2]; (I) intervention: high-flow nasal cannula (HFNC) strategies; (C) Comparison: continuous positive airway pressure (CPAP) and conventional oxygen therapy (COT), which includes methods such as nasal cannula, nasal prongs, nasal catheters, or masks; (O) outcome: the primary outcome: treatment failure and intubation rates, secondary outcome: duration of oxygen therapy, PICU length of stay (LOS), hospital LOS and hospital mortality rate; (S) study type: Only RCTs that provide direct comparisons between HFNC and CPAP or COT were included.

Conversely, studies excluded if they were non-randomized, such as cohort studies, cross-sectional studies, case reports, or review articles, including systematic reviews without original data. Studies that failed to provide data on the outcomes of interest or that did not include comparative findings between HFNC and CPAP or COT. Additionally, studies that involve adult populations > 18 years or those not diagnosed with ARF, will not be considered for inclusion.

### Data collection

In order to improve the efficiency of managing search results and screening for relevant studies, the studies were transferred to EndNote software (version: 20.2.1). This process involved removing duplicate entries and ensuring proper referencing. Initially, two independent researchers (E.M. & S.A.) screened titles and abstracts to identify studies that addressed the research questions. Following this, the same researchers carefully evaluated full-text articles to select studies that met the inclusion criteria. Articles meeting the criteria were included, while those that did not were excluded. To maintain consistency, inter-rater reliability between the researchers was assessed, with any discrepancies resolved through discussion or consultation with a third researcher. The three evaluators reached an agreement with a high Kendall’s coefficient of agreement (*r* = 0.92; *P* < 0.001). The final search results were illustrated in a flow diagram according to the guidelines set out in the PRISMA statement.

### Quality appraisal

RCTs identified for retrieval were assessed by two independent authors (E.M. & S.A.) for methodological quality before inclusion in the review using a standardized critical appraisal tool. Any divergence was resolved by mutual consensus in the presence of a third investigator. Risk of bias was assessed by the Cochrane Risk of Bias (ROB) Tool for RCTs [21]. Each study was assessed for (1) random sequence generation (selection bias); (2) allocation concealment (selection bias); (3) blinding of participants and personnel (performance bias); (4) blinding of related outcomes assessment (detection bias); (5) incomplete outcome data (attrition bias); (6) selective reporting (reporting bias); and (7) other biases.

### Data extraction

Two researchers independently reviewed the full texts of all eligible studies and extracted data using a pre-established checklist, which was then recorded in an electronic file (Excel sheets). The extracted information included the study characteristics (e.g., first author’s name, publication year, country, study design and setting), participant demographics (e.g., cause of ARF; bronchiolitis, pneumonia or asthma), intervention details (e.g., sample size), comparator details (e.g., type of control group; CPAP or COT, sample size), outcome measures includes treatment failure (treatment failure was determined by clinical signs including respiratory rate, heart rate, pulse oximetry, and a need to escalate treatment. The criteria for treatment failure varied slightly among included studies, but presented by frequency and percent %), intubation rate (intubation rates was determined by need more NIV mechanical or even invasive mechanical ventilator that presented by frequency and percent %), duration of oxygen therapy (in days), PICU LOS (in days) hospital LOS (in days) and hospital mortality (%), and key findings.

### Statistical analysis

Quality assessment, data processing, and graphical representations (including all plots) were conducted using RevMan 5.4 software and R software (Version 4.3.1). For dichotomous variables (e.g., treatment failure, intubation rate, and hospital mortality), the odds ratio (OR) with a 95% confidence interval (CI) was calculated. For continuous variables (e.g., duration of oxygen therapy, PICU LOS and hospital LOS), the weighted mean difference (MD) with a 95% CI was used as the effect measure.

Study heterogeneity was assessed using Cochran’s Q test (χ²) and the I^2^ statistic. Given the anticipated clinical and methodological diversity across the included trials (e.g., variations in patient populations, baseline disease severity, and specific intervention protocols), we a priori selected the random-effects model (DerSimonian and Laird method) for all meta-analyses, regardless of the I^2^ value. This approach is strongly recommended in the literature, as it accounts for both within-study and between-study variance, providing more conservative and generalizable effect estimates when synthesizing data from published clinical trials [22].

Funnel plots, Begg’s test and Egger’s test were generated to evaluate potential publication bias among the included studies. Additionally, subgroup analyses were conducted to investigate potential sources of heterogeneity, focusing on the etiology of ARF (bronchiolitis, pneumonia, asthma). The significance level for the meta-analysis was set at α = 0.05, with P-values less than 0.05 considered statistically significant.

## Results

### Search outcomes

**Fig. 1:**
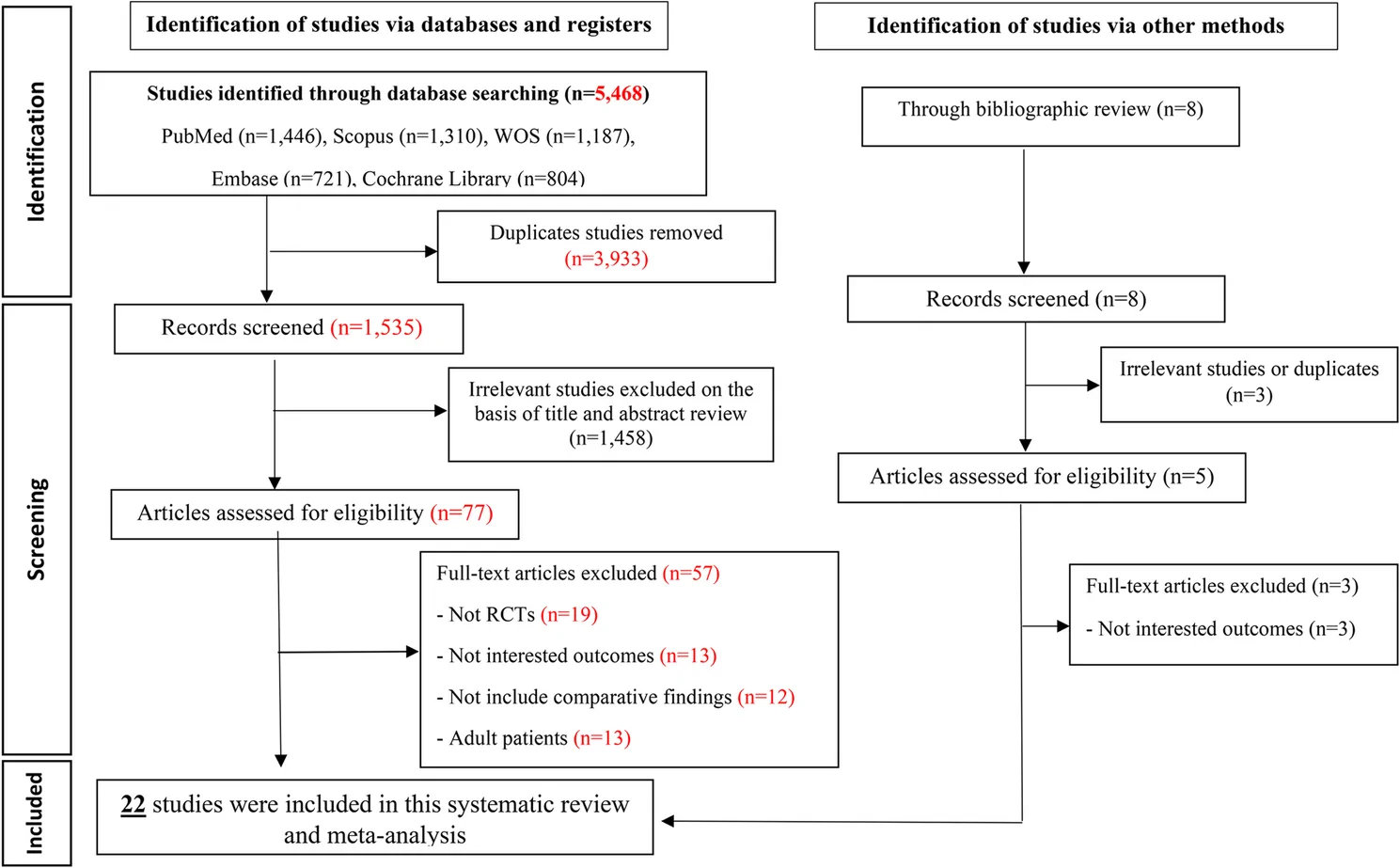
The literature searches results and the screening process based on PRISMA 2020 flowchart

We conducted a comprehensive search across several databases: PubMed (*n* = 1,446), Scopus (*n* = 1,310), Web of Science (*n* = 1,187), Embase (*n* = 721), and the Cochrane Library (*n* = 804), which resulted in a total of 5,468 articles. Additionally, we identified 8 more articles through bibliographic references, bringing the total to 5,476 articles. After removing duplicates (*n* = 3,936) and screening the titles and abstracts of the remaining records (*n* = 1,540), we evaluated 82 full-text articles for eligibility. Of these, 60 articles were excluded based on our inclusion and exclusion criteria: 19 were not RCTs, 13 involved adult patients, 16 did not address the outcomes of interest (e.g.; compared HFNC to NIV/BiPAP or evaluated post-extubation/weaning protocols rather than ARF), and 12 not include comparative findings. Ultimately, 22 RCTs met the inclusion criteria and were incorporated into our analysis (Fig. 1).

### Studies characteristics

This analysis included a total of 22 RCTs that involved 6,641 children [12–15, 23–40]. The participants were assigned to three different treatments: HFNC (*n* = 3,328), COT (*n* = 2,833), and CPAP (*n* = 421). Among the 22 RCTs, 15 were conducted at a single center [12, 14, 25–28, 30–33, 36–40], while 7 were multicenter studies [13, 15, 23, 24, 29, 34, 35]. Twelve of these studies compared HFNC with COT [13, 23, 24, 26, 27, 29, 31–34, 37, 39], nine compared HFNC with CPAP [12, 14, 15, 25, 28, 30, 35, 36, 38], and one study compared HFNC with both COT and CPAP [40]. The ARF examined in these trials caused by acute bronchiolitis in 12 trials [12, 14, 15, 25–29, 33, 34, 36, 39], pneumonia in 3 trials [13, 30, 40], and asthma in 2 trials [31, 32]. However, five trials only mentioned children with acute ARF [23, 24, 35, 37, 38]. Geographically, the trials were distributed as follows: 11 were conducted in Asia [12, 23, 24, 26, 30, 33, 34, 36, 37, 39, 40], 5 in Europe [15, 25, 29, 32, 38], 2 in South America [28, 31], 3 in Africa [13, 14, 27], and 1 in the United Kingdom [35]. The baseline characteristics of the enrolled studies are presented in Table 1.

**Table 1:** Characteristics of the included studies

Author, country, year (Ref)	Type of study	Setting	Intervention vs. control	Sample size	Age	Respiratory disorders	Outcomes	Findings
Malini et al., India [12]	Open-label RCT (Single-center)	PICU	HFNC vs. CPAP	T: 118 I: 59 C: 59	I: 7 (3–11) C: 4 (3–8) months	Moderate to severe acute bronchiolitis	• Treatment failure 14 (23.7%) vs. 25 (42.4%), *P* = 0.031* OR: 0.43, 95%CI: 0.2–0.93, *P* = 0.031* • Intubation rate 9 (15.3%) vs. 23 (39%), *P* = 0.004* OR: 0.28, 95%CI: 0.12–0.68, *P* = 0.004* • Oxygen therapy (days) 4 (3–6) vs. 3 (3–5), *P* = 0.012* • Hospital LOS (days) 6 (5-8.5) vs. 5 (4–7), *P* = 0.021*	In children aged one to 23 months with moderate to severe acute bronchiolitis, the use of HFNC therapy as opposed to CPAP for early respiratory support was associated with a significantly lower treatment failure rate and a substantially reduced risk of escalation to mechanical ventilation. However, HFNC was linked to a longer duration of oxygen therapy and a longer hospital length of stay compared to CPAP.
Franklin et al., Australia and New Zealand [23]	RCT (multicenter)	GW and PICU	HFNC vs. COT	T:1517 I: 753 C: 764	I: 1.9 (1.4-3) C: 1.9 (1.4–2.9) years	Acute respiratory disorders	• Intubation rate 11 (11.7%) vs. 6 (11.3%) OR: 1.04, 95%CI: 0.37–2.91, *P* = 0.94 • Hospital LOS (days) 1.77 (1.03–2.8) vs. 1.5 (0.85–2.44), *P* < 0.001* • Oxygen therapy (days) 1.07 (0.50–2.06) vs. 0.75 (0.35–1.61), *P* < 0.001*	HFNC as the initial primary therapy in children aged 1 to 4 years with acute respiratory disease did not significantly reduce the intubation rate compared to COT. However, HFNC was associated with a statistically significant increase in both hospital LOS and duration of oxygen therapy compared to conventional oxygen therapy.
Maitland et al., (a) Ugandan, Kenya [13]	Open-label RCT (multicenter)	GW	HFNC vs. COT	T: 388 I: 194 C: 194	I: 7 (2–21) C: 7 (2–16) months	Severe pneumonia (Saturations SpO2 < 80%)	• Treatment failure 33 (17.1%) vs. 44 (22.8%) • OR: 0.64, 95%CI: 0.41–0.99, *P* < 0.05* • Hospital LOS (days) 6.3 ± 5.6 vs. 6.2 ± 6.1 MD: 0.1, 95%CI: -0.95 to 1.15 • Hospital mortality 18 (9.3%) vs. 26 (13.4%) OR: 0.70, 95%CI: 0.06–0.93, *P* = 0.046*	In children with severe pneumonia, HFNC was associated with a significantly lower risk of treatment failure and hospital mortality compared to COT. However, there was no significant difference in hospital LOS between the two groups.
Maitland et al., (b) Ugandan, Kenya [13]	Open-labelled RCT (multicenter)	GW	HFNC vs. COT	T: 727 I: 363 C: 364	I: 9 (4–24) C: 9 (4–22) months	Moderate pneumonia (Saturations SpO2 80 to < 92%)	• Treatment failure 9 (2.5%) vs. 17 (4.7%) • OR: 0.55, 95%CI: 0.33–0.91, *P* < 0.05* • Hospital LOS (days) 5.3 ± 10.4 vs. 4.9 ± 3.7 MD: 0.4, 95%CI: -0.74 to 1.54, *P* = 0.49 • Hospital mortality 4 (1.1%) vs. 9 (2.5%) OR: 0.60, 95%CI: 0.33–1.06, *P* = 0.076	HFNC was associated with a significantly lower risk of treatment failure compared to COT. However, there was no significant difference in hospital LOS. While HFNC showed a trend toward reduced hospital mortality, this difference did not reach statistical significance. HFNC demonstrates potential benefits in reducing treatment failure without significantly impacting the duration of hospitalization.
Franklin et al., Australia and New Zealand [24]	open-labelled RCT (multicenter)	ED and GW	HFNC vs. COT	T: 563 I: 283 C: 280	I: 2.4 (1.5–4.1) C: 2.3 (1.5–3.7) years	Acute respiratory disorders	• Treatment failure 32 (11.7%) vs. 50 (18.1%) OR: 0.62, 95%CI: 0.38-1.00, *P* > 0.05 • Oxygen therapy (days) 0.8 (0.3–1.8) vs. 0.9 (0.4–1.8) • Hospital LOS (days) 2 (1.2-3) vs. 1.9 (1–2) • PICU LOS (*N* = 48) (days) 1.4 (0.9–1.8) vs. 1.7 (0.7–2.6)	No significant differences were observed between the two groups of children who received HFNC and COT in terms of PICU admissions, treatment failure, length of hospital stay, or PICU stay.
Borgi et al., Tunisia [14]	RCT (Single-center)	PICU	HFNC vs. CPAP	T: 255 I: 130 C: 125	I: 53.5 (30.06) C: 48.6 (32.61) days	Severe bronchiolitis	• Treatment failure 64 (49.2%) vs. 37 (29.6%), *P* = 0.001* OR: 2.32, 95%CI: 1.41–3.82, *P* = 0.001 • Intubation rate 29 (22.3%) vs. 37 (29.6%), *P* = 0.25 OR: 0.68, 95%CI: 0.39–1.20, *P* = 0.25 • Hospital LOS (days) 5.9 ± 4.1 vs. 6.7 ± 5.6, *P* = 0.16 MD: -0.8, 95%CI: -2.01 to 0.41	CPAP is the optimal first-line respiratory support for severe bronchiolitis, as it is associated with a significantly lower treatment failure rate compared to HFNC. However, no significant differences were observed in intubation rates or hospital length of stay between the two groups. When HFNC fails, switching to CPAP may help prevent intubation in a substantial proportion of cases.
Vahlkvist et al., Denmark [25]	RCT (Single-center)	ED	HFNC vs. CPAP	T: 50 I: 22 C: 28	I: 2.1 (0.5–8.8) C: 2.8 (0.3–11.3) months	Bronchiolitis	• Treatment failure 2 (9.1%) vs. 4 (14.3%) OR: 0.60, 95%CI: 0.10–3.63, *P* = 0.58 • Intubation rate 2 (9.1%) vs. 4 (14.3%) OR: 0.60, 95%CI: 0.10–3.63, *P* = 0.58	No significant differences were observed between the two groups in terms of treatment failure or intubation rates
Türe et al., Turkey [26]	RCT (Single-center)	ED	HFNC vs. COT	T: 75 I: 37 C: 38	I: 8 (3–24) C: 9.5 (2–24) months	Severe bronchiolitis	• Hospital LOS (days) 1.29 ± 0.5 vs. 1.84 ± 0.66, *P* = 0.001* MD: -0.55, 95%CI: -0.79 to -0.31, *P* = 0.001* • Oxygen therapy (days) 0.71 ± 0.4 vs. 1.05 ± 0.44, *P* = 0.001 MD: -0.34, 95%CI: -0.53 to -0.15, *P* = 0.001*	HFNC significantly shortens length of hospital stays and duration of oxygen therapy compared with standard oxygen.
Murphy et al., South Africa [27]	RCT (Single-center)	PICU	HFNC vs. COT	T: 28 I: 15 C: 13	I: 4 (2–11) C: 5 (3–9) months	Moderate-severe viral bronchiolitis	• Intubation rate 3 (20%) vs. 6 (46%), *P* = 0.139 OR: 0.33, 95%CI: 0.08–1.38, *P* = 0.139 • Hospital LOS (days) 8 (4–9) vs. 8 (4–9), *P* = 0.44 • Oxygen therapy (days) 5.5 (3.2–6.7) vs. 6 (3–7), *P* = 0.7	There was a significant improvement in respiratory distress, in infants who received HFNC therapy. Additionally, there was also a reduction in heart rate in the HFHO group as well as a trend to lower intubation rates. However, no significant differences were observed between the two groups in terms of hospital LOS, duration of oxygen therapy or need to intubation.
Cesar et al., Brazil [28]	RCT (Single-center)	PICU	HFNC vs. CPAP	T: 63 I: 35 C: 28	I: 3.4 (1.4–5.4) C: 2.4 (0.9–3.3) month	Moderate-severe bronchiolitis	• Treatment failure 13 (37.1%) vs. 10 (35.7%) OR: 1.04, 95%CI: 0.56–1.93, *P* = 0.88 • Intubation rate 7 (20%) vs. 3 (11%) OR: 1.94, 95%CI: 0.57–6.69, *P* = 0.49 • Hospital LOS (days) 9 (7–12) vs. 8 (7–11) • PICU LOS (days) 5 (4–8) vs. 5 (4–7)	The results indicate that the rate of treatment failure, including the need for intubation, was similar between the CPAP and HFNC groups. Additionally, both hospital LOS and PICU LOS did not significantly differ between the two treatment groups. This pilot study suggests that HFNC was comparable to CPAP in terms of treatment failure and clinical outcomes for the studied population.
Durand et al., France [29]	RCT (multicenter)	ED	HFNC vs. COT	T: 268 I:133 C: 135	I: 68 ± 48 C: 65 ± 46 days	Moderate bronchiolitis	• Treatment failure 19 (14%) vs. 27 (20%) OR: 0.66, 95%CI: 0.35–1.26, *P* = 0.21 • Oxygen therapy (days) 1.7 ± 1.7 vs. 2.5 ± 2 MD: -0.80, 95%CI: -1.2 to -0.3, *P* < 0.05* • Hospital LOS (days) 4.4 ± 2.4 vs. 3.8 ± 2.7 MD: 0.6, 95%CI: -0.04 to 1.2	In children with moderate bronchiolitis, there was no evidence of a lower rate of escalating respiratory support or reduced PICU admissions among those receiving HFNC therapy compared to the alternative treatment. However, HFNC therapy was associated with a shorter duration of oxygen therapy. Hospital LOS did not significantly differ between the two groups.
Liu et al., China [30]	RCT (Single-center)	ED	HFNC vs. CPAP	T: 84 I: 43 C: 41	I: 3 (2–11) C: 4 (1–11) months	Mild to moderate pneumonia	• Intubation rate 6 (14%) vs. 4 (10%) OR: 1.47, 95%CI: 0.45–4.72, *P* = 0.553 • Oxygen therapy (days) 2 (2–3) vs. 3 (2–3) • Hospital LOS (days) 8 (7–9) vs. 8 (7–9) *P* = 0.461	HFNC is an effective and safe initial respiratory support treatment in children < 2 years with mild to moderate respiratory failure due to pneumonia, and the incidence of intubation and death is very low; concurrently, the comfort and tolerance of HFNC are better.
Benítez et al., Paraguay [31]	Open-label RCT (Single-center)	ED	HFNC vs. COT	T: 65 I: 32 C: 33	I: 5 (2–14) C: 4 (2–14) months	Severe asthma	• Hospital LOS (days) 1.03 ± 0.51 vs. 1.004 ± 0.62, MD: 0.03, 95%CI: -0.44 to 0.49), *P* = 0.598	The incorporation of HFNC oxygen therapy in the treatment of patients with asthmatic crises in the Pediatric ED did not show clinical benefits nor did it diminish the stay time.
Ballestero et al., Spain [32]	RCT (Single-center)	ED	HFNC vs. COT	T: 62 I: 30 C: 32	I: 3 (1.7-6) C: 3 (2–6) years	Moderate-to-severe asthma	• Treatment failure 8 (26.7%) vs. 9 (28.1%) OR: 0.92, 95%CI: 0.39–2.15, *P* = 0.87 • PICU LOS (min) 48 (24–48) vs. 48 (24-64.8) *P* > 0.05	HFNC appears to be superior to COT for reducing respiratory distress within the first 2 h of treatment as a first-line treatment. However, between the group’s differences in treatment failure or PICU LOS were not significant.
Ergul et al., Turkey [33]	RCT (Single-center)	PICU	HFNC vs. COT	T: 60 I: 30 C: 30	I: 11.1 (7–20) C: 10.1 (6–11) months	Moderate-to-severe bronchiolitis	• Treatment failure 0 (0) vs. 7 (23.3), *P* = 0.011* OR: 0.14, 95%CI: 0.02–0.78, *P* = 0.017* • PICU LOS (days) 3 (2–3) vs. 4 (3–5), *P* = 0.001* • Hospital LOS (days) 4 (3–4) vs. 5 (4–6), *P* = 0.001*	HFNC use decreased the treatment failure rate and the duration of both oxygen therapy and ICU treatment compared to the COT, which implies that an HFNC should be the first choice for treating patients admitted to the ICU with severe bronchiolitis.
Franklin et al., Australia and New Zealand [34]	RCT (multicenter)	ED and GW	HFNC vs. COT	T: 1472 I: 739 C: 733	I: 5.76 ± 3.54 C: I: 6.1 ± 3.44 months	Moderate-to-severe bronchiolitis	• Treatment failure 87 (12%) vs. 167 (23%) OR: 0.48, 95%CI: 0.37–0.62, *P* < 0.001* • Hospital LOS (days) 3.12 ± 2.43 vs. 2.94 ± 2.73 MD: 0.18, 95%CI: -0.09 to 0.44), *P* = 0.19 • PICU LOS (days) 2.63 ± 1.70 vs. 2.72 ± 2.31 MD: -0.09, 95%CI: -0.74 to 0.55), *P* = 0.78 • Oxygen therapy (days) 1.81 ± 2.18 vs. 1.87 ± 2.09 MD: -0.06, 95%CI: -0.28 to 0.16, *P* = 0.61	Treatment failure was significantly lower in the children who received HFNC compare to COT. However, no significant differences were observed in the hospital LOS, PICU LOS or the duration of oxygen therapy.
Ramnarayan et al., UK [35]	RCT (multicenter)	PICU	HFNC vs. CPAP	T: 29 I: 16 C: 13	I: 1.5 (0.6-7) C: 0.8 (0.7-1) years	Acute respiratory disorders	• Intubation rate 6 (37.5%) vs. 2 (15.4%) OR: 3.23, 95%CI: 0.76–13.61, *P* = 0.13 • Hospital LOS (days) 24.1 ± 26.1 vs. 31.9 ± 29.1 MD: -7.8, 95%CI: -28.9 to 13.3, *P* = 0.556 • PICU LOS (days) 6.7 ± 5.8 vs. 5.9 ± 5.2 MD: 0.7, 95%CI: -3.5 to 5 *P* = 0.696 • Hospital mortality 2 (12.5%) vs. 1 (7.7%) OR: 1.63, 95%CI: 0.17–15.9, *P* = 0.727	HFNC and CPAP showed no statistically significant differences in intubation rates, hospital or PICU LOS, or hospital mortality in children with respiratory disorders. However, trends suggest HFNC may be associated with a higher intubation rate and shorter hospital LOS, though further studies with larger sample sizes are needed to confirm these findings.
Sarkar et al., India [36]	Open-label RCT (Single-center)	PICU	HFNC vs. CPAP	T: 31 I: 15 C: 16	I: 4.06 ± 2.92 C: 2.81 ± 1.03 months	Severe bronchiolitis	• Intubation rate 1 (6.7%) vs. 1 (6.2%) OR: 0.93, 95%CI: 0.05–6.35, *P* = 0.29 • Treatment failure 1 (6.7%) vs. 1 (6.2%) OR: 0.93, 95%CI: 0.05–6.35, *P* = 0.29 • Oxygen therapy (days) 3.6 ± 0.63 vs. 3.8 ± 0.8 MD: -0.2, 95%CI: -0.72 to 0.32, *P* = 0.33 • PICU LOS (days) 5 ± 1.6 vs. 5 ± 1.78 MD: 0, 95%CI: -1.12 to 1.12, *P* = 0.105	HFNC and CPAP demonstrated similar outcomes in terms of intubation rates, treatment failure, oxygen therapy duration, and PICU LOS for children with severe bronchiolitis. Neither intervention showed superiority, suggesting both are equally effective in managing severe bronchiolitis in this population.
Sitthikarnkha et al., Thailand [37]	RCT (Single-center)	PICU	HFNC vs. COT	T: 98 I: 49 C: 49	I: 16.7 ± 12.6 C: 20.7 ± 14.0 months	Acute respiratory distress	• Treatment failure 4 (8.2%) vs. 10 (20.4%), *P* = 0.09 OR: 0.15, 95% CI: 0.03–0.66, *P* = 0.01* • Intubation rate 4 (8.2%) vs. 7 (14.3%), *P* = 0.12 OR: 0.53, 95% CI: 0.15–1.95, *P* = 0.098	There was an 85% reduction in the odds of treatment failure in HFNC therapy group. Most children in HFNC therapy group had significant improvement in clinical respiratory score, heart rate, and respiratory rate compared with COT group.
Vitaliti et al., Italy [38]	RCT (Single-center)	ED	HFNC vs. CPAP	T: 40 I: 20 C: 20	I: 21.5 ± 2.6 C: 5.20 ± 3.3 months	Mild to-moderate respiratory distress	• Treatment failure 1 (5%) vs. 0 (0%), *P* = 0.10 OR: 1.32, 95% CI: 0.01–4.66, *P* = 0.120 • Hospital LOS (days) 13.1 ± 1.32 vs. 4.9 ± 1.1, *P* < 0.001 MD: 8.2, 95%CI: 7.45–8.95, *P* < 0.001	while HFNC and CPAP showed no significant difference in treatment failure, HFNC was associated with a significantly longer hospital stay in children with respiratory conditions.
Milési et al. France [15]	RCT (multicenter)	PICU	HFNC vs. CPAP	T: 142 I: 71 C: 71	I: 42 ± 39 C: 38 ± 32 Days	Moderate to severe acute viral bronchiolitis	• Treatment failure 36 (50.7%) vs. 22 (31%), *P* = 0.001* OR: 2.3, 95%CI: 1.16–4.55, *P* = 0.001* • Intubation rate 5 (6.9%) vs. 3 (4.2%), *P* = 0.72 0R: 1.72, 95%CI: 0.4–7.5, *P* > 0.72 • PICU LOS (days) 6.2 ± 1.6 vs. 7.5 ± 1.3, *P* = 0.44 MD: -1.3, 95%CI: -4.6 to 2.03, *P* > 0.44	The results showed that the HFNC had a significantly higher risk of treatment failure compared to CPAP. However, no significant differences were observed in intubation rates or PICU length of stay between two groups.
Kepreotes et al., Australia [39]	Open-label RCT (Single-center)	ED	HFNC vs. COT	T: 202 I: 101 C: 101	I: 6 (3–10) C: 5 (3–10) months	Moderate bronchiolitis	• Treatment failure 14 (14%) vs. 33 (33%), *P* = 0.0016* OR: 0.33, 95%CI: 0.16–0.66, *P* = 0.0016* • PICU LOS (days) 2 (1–3) vs. 2 (1–3), *P* = 0.99 • Oxygen therapy (days) 0.83 (0.71–1.42) vs. 1 (0.75–1.17)	HFNC demonstrated a significant advantage in reducing treatment failure compared to conventional oxygen therapy, while no differences were observed in PICU LOS or oxygen therapy duration.
Chisti et al., Bangladesh [40]	Open-label RCT (Single-center)	PICU	HFNC vs. CPAP and HFNC vs. COT	T: 225 HFNC: 79 CPAP: 79 COT: 67	H: 6.5 (4–13) CPAP: 9 (3–14) COT: 7.5 (4–11)	Severe pneumonia	• Treatment failure HFNC vs. CPAP 10 (13%) vs. 5 (6%) OR: 2.23, 95%CI: 0.73–6.84, *P* = 0.16 HFNC vs. COT 10 (13%) vs. 16 (24%) OR: 0.46, 95% CI: 0.2–1.07, *P* = 0.07 • Intubation rate HFNC vs. CPAP 10 (13%) vs. 5 (6%) OR: 2.23, 95%CI: 0.73–6.84, *P* = 0.16 HFNC vs. COT 10 (13%) vs. 11 (16%) OR: 0.76, 95% CI: 0.31–1.89, *P* = 0.56 • Hospital mortality HFNC vs. CPAP 10 (13%) vs. 3 (4%) OR: 3.5, 95% CI: 0.93–13.2, *P* = 0.06 HFNC vs. COT 10 (13%) vs. 10 (15%) OR: 0.82, 95% CI: 0.32–2.1, *P* = 0.68 • Hospital LOS (days) HFNC vs. CPAP 5 (3–7) vs. 5 (3–7), *P* = 0.493 HFNC vs. COT 5 (3–7) vs. 4 (3–7)	While HFNC showed some trends in treatment failure and hospital mortality compared to CPAP and COT, none of these differences reached statistical significance. No significant differences were observed in intubation rates or hospital LOS.

*HFNC *High-Flow Nasal Cannula, *CPAP *Continuous Positive Airway Pressure, *COT *Conventional Oxygen Therapy, *PICU *Pediatric Intensive Care Unit, *LOS *Length of Stay, *OR *Odds Ratio, *CI *Confidence Interval, *MD* Mean Difference, *RCT *Randomized Controlled Trial

### Results of quality assessment

**Fig. 2:**
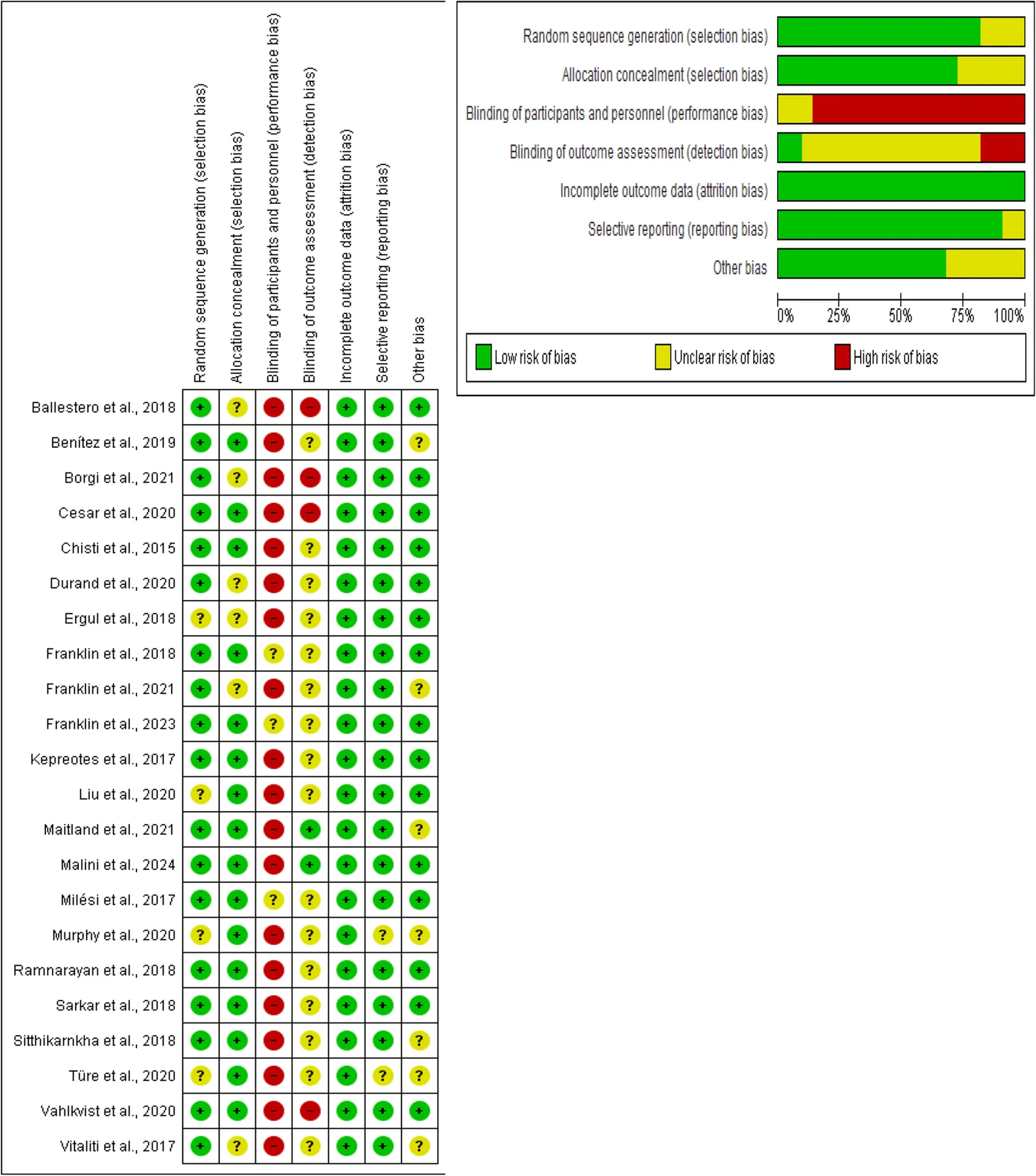
Risk of bias assessment according to the Cochrane risk-of-bias tool for included studies

According to the Risk of Bias (ROB) tool (Fig. 2), the majority of studies demonstrated low risks associated with selection and allocation concealment biases [12, 13, 15, 23–25, 28, 31, 33–37, 39, 40]. However, blinding of clinicians, researchers, or patients was not feasible for any study due to the audible and visible differences in oxygen delivery devices. While attrition and reporting biases were generally assessed as low risk, there were instances of unclear risk for reporting bias, particularly in studies by Türe et al. [26] and Murphy et al. [27]. Additionally, although many sources of bias were rated low, several studies indicated unclear risk [13, 24, 26, 27, 31, 37, 38]. This highlights the necessity of interpreting the findings with caution, as these biases could undermine the validity of the studies. Ultimately, since no trials achieved a low risk across all domains, we classified all trials as being at high risk of bias.

### Meta-analysis of primary outcomes

#### Treatment failure

**Fig. 3:**
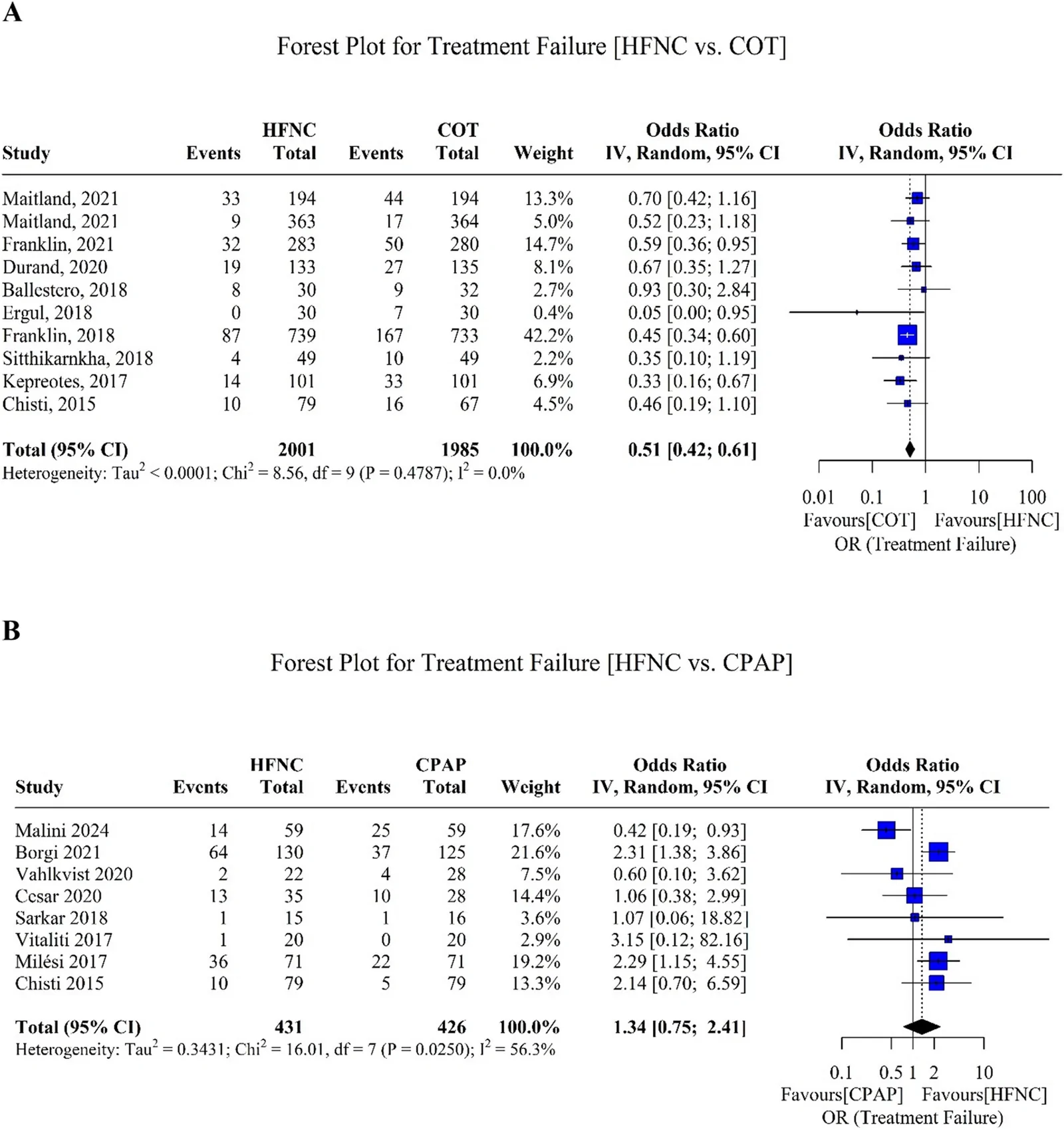
Forest plot for the pairwise comparison of treatment failure; (**A**) high-flow nasal cannula (HFNC) therapy vs. conventional oxygen therapy (COT), and (**B**) high-flow nasal cannula (HFNC) therapy vs. continuous positive airway pressure (CPAP)

Nine trials evaluated the effects of HFNC compared to COT on treatment failure in a total of 3,986 children [13, 24, 29, 32–34, 37, 39, 40], while eight trials assessed HFNC versus CPAP in 857 children [12, 14, 15, 25, 28, 36, 38, 40]. The results indicated that HFNC therapy significantly reduced the risk of treatment failure compared to COT (OR: 0.509, 95% CI: 0.42–0.61, *P* < 0.001), with no significant heterogeneity observed among these studies (I²: 0%, *P* = 0.479) (Fig. 3A). In contrast, no significant difference was noted in treatment failure rates between HFNC and CPAP (OR: 1.343, 95% CI: 0.75–2.4, *P* = 0.323), and a notable level of heterogeneity was present in these studies (I²: 56.3%, *P* = 0.025) (Fig. 3B).

#### Intubation rate

**Fig. 4:**
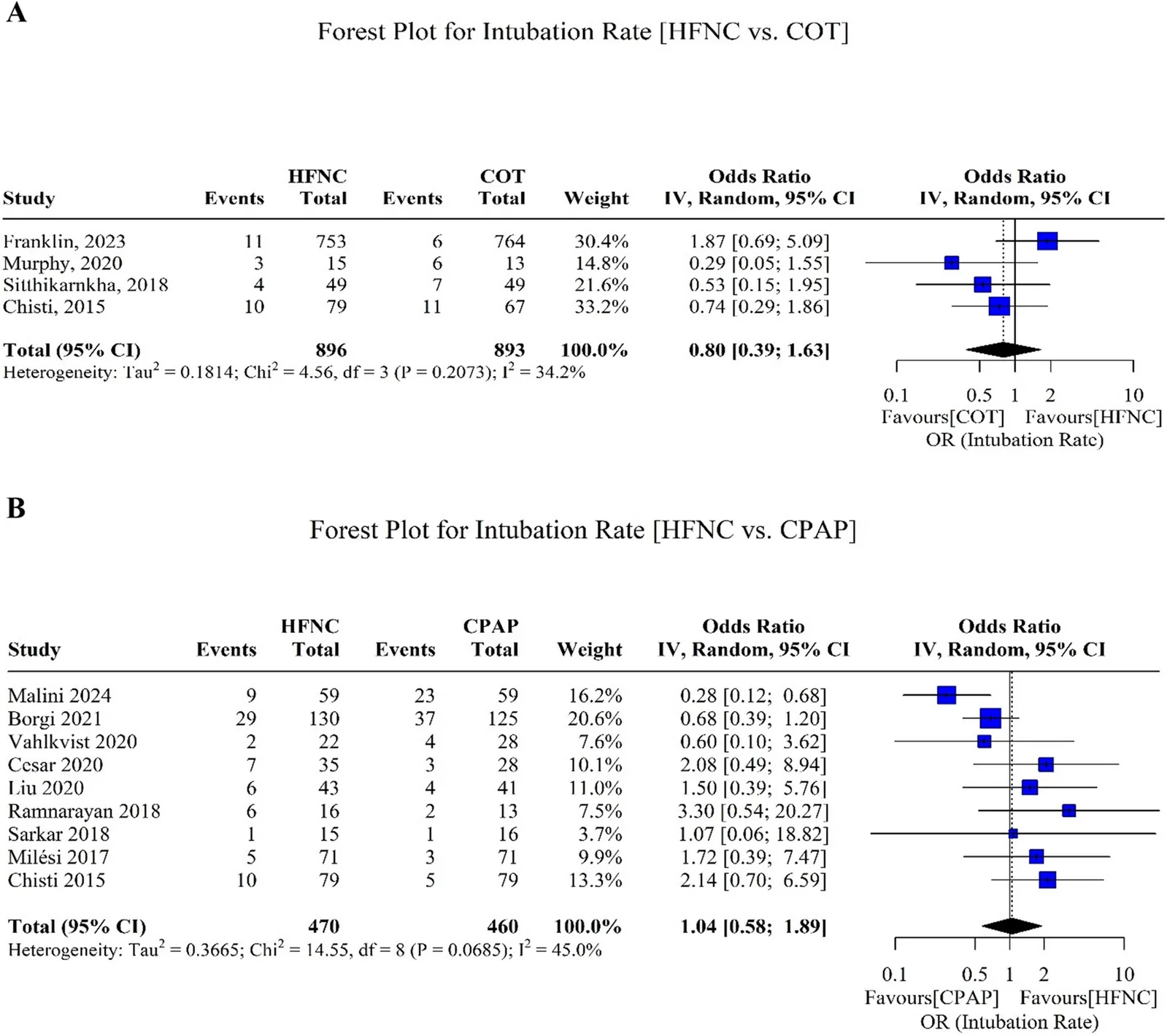
Forest plot for the pairwise comparison of intubation rate; (**A**) high-flow nasal cannula (HFNC) therapy vs. conventional oxygen therapy (COT), and (**B**) high-flow nasal cannula (HFNC) therapy vs. continuous positive airway pressure (CPAP)

Four trials examined the impact of HFNC compared to COT on intubation rates in a total of 1,789 children [23, 27, 37, 40]. Additionally, nine trials investigated HFNC versus CPAP in 930 children [12, 14, 15, 25, 28, 30, 35, 36, 40]. The results showed no significant differences in intubation rates between HFNC and COT (OR: 0.796, 95% CI: 0.39–1.63, *P* = 0.533) or between HFNC and CPAP (OR: 1.044, 95% CI: 0.58–1.89, *P* = 0.887). Additionally, no significant heterogeneity was observed among the studies regarding intubation rates for COT (I²: 34.2%, *P* = 0.207) or for CPAP (I²: 45%, *P* = 0.068) (Fig. 4A and B).

### Meta-analysis of secondary outcomes

#### Duration oxygen therapy

**Fig. 5:**
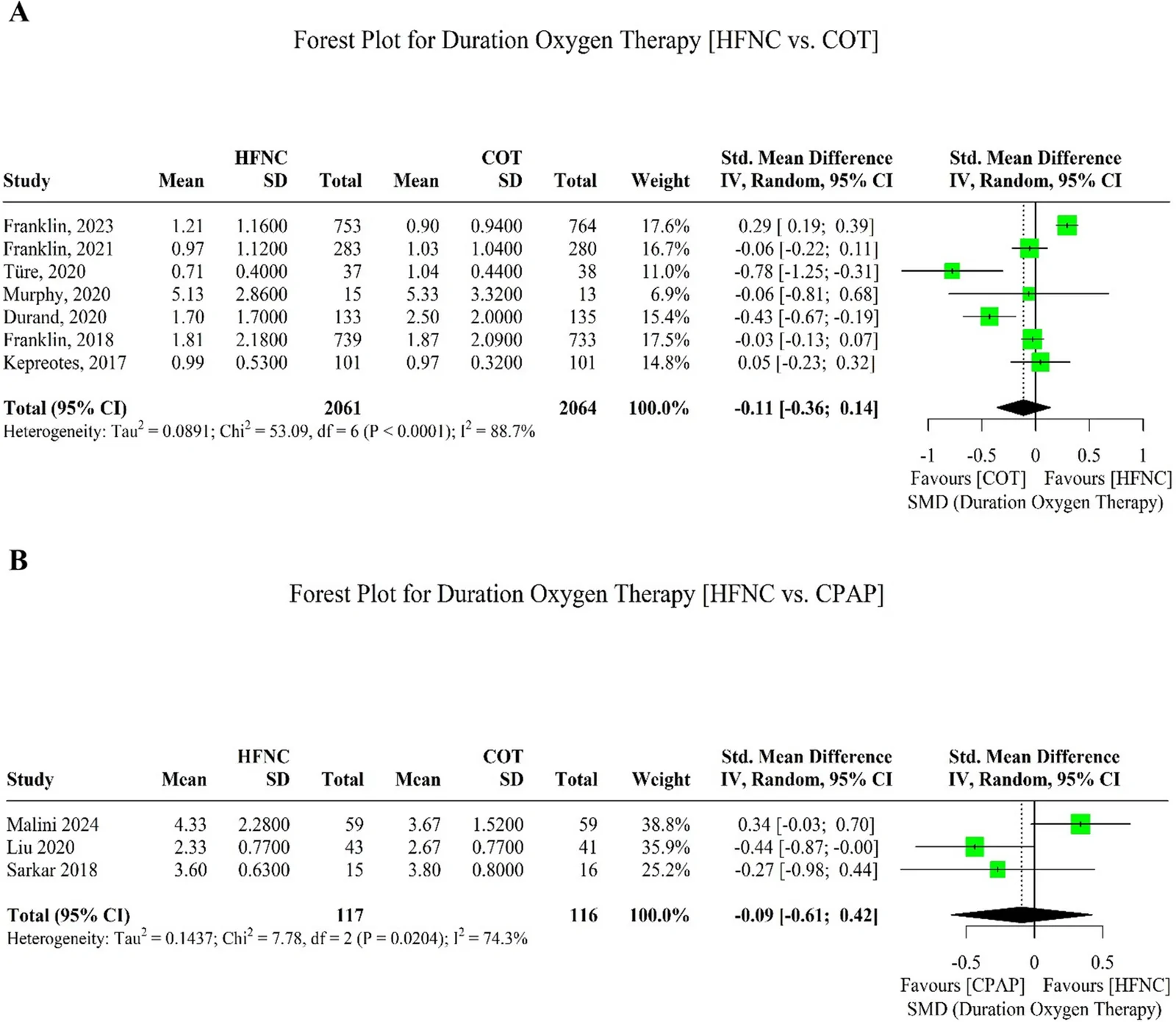
Forest plot for the pairwise comparison of duration of oxygen therapy (days); (**A**) high-flow nasal cannula (HFNC) therapy vs. conventional oxygen therapy (COT), and (**B**) high-flow nasal cannula (HFNC) therapy vs. continuous positive airway pressure (CPAP)

Seven trials assessed the effect of HFNC compared to COT on the duration of oxygen therapy, involving a total of 4,125 children [23, 24, 26, 27, 29, 34, 39]. In contrast, only three trials compared HFNC to CPAP in 233 children [12, 30, 36]. The findings revealed no significant differences in the duration of oxygen therapy between HFNC and COT (MD: -0.112, 95% CI: -0.36 to 0.14, *P* = 0.381) or between HFNC and CPAP (MD: -0.093, 95% CI: -0.061 to 0.42, *P* = 0.726). Furthermore, there was a considerable degree of heterogeneity among the studies regarding the duration of oxygen therapy for COT (I²: 88.7%, *P* < 0.001) and for CPAP (I²: 74.3%, *P* = 0.021) (Fig. 5A and B).

#### PICU LOS

**Fig. 6:**
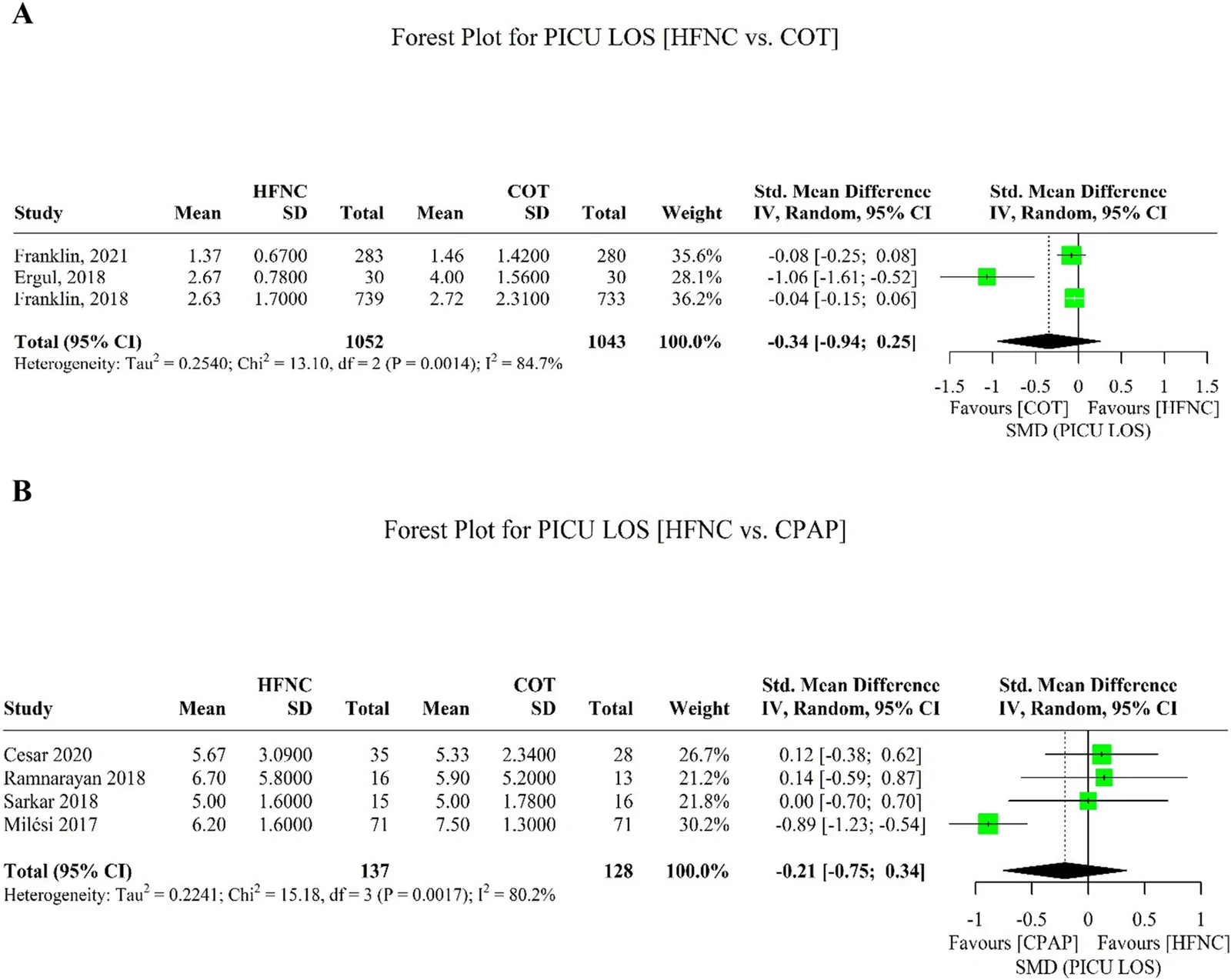
Forest plot for the pairwise comparison of PICU LOS (days); (**A**) high-flow nasal cannula (HFNC) therapy vs. conventional oxygen therapy (COT), and (**B**) high-flow nasal cannula (HFNC) therapy vs. continuous positive airway pressure (CPAP)

Three trials evaluated the effect of HFNC compared to COT on the LOS in the PICU, encompassing a total of 2,095 children [24, 33, 34]. Additionally, four trials compared HFNC to CPAP in 265 children [15, 28, 35, 36]. The results indicated no significant differences in PICU LOS between HFNC and COT (MD: -0.344, 95% CI: -0.94 to 0.25, *P* = 0.261) or between HFNC and CPAP (MD: -0.206, 95% CI: -0.75 to 0.34, *P* = 0.458). Moreover, there was a substantial level of heterogeneity among studies regarding PICU LOS for COT (I²: 84.7%, *P* < 0.001) and for CPAP (I²: 80.2%, *P* = 0.001) (Fig. 6A and B).

#### Hospital LOS

**Fig. 7:**
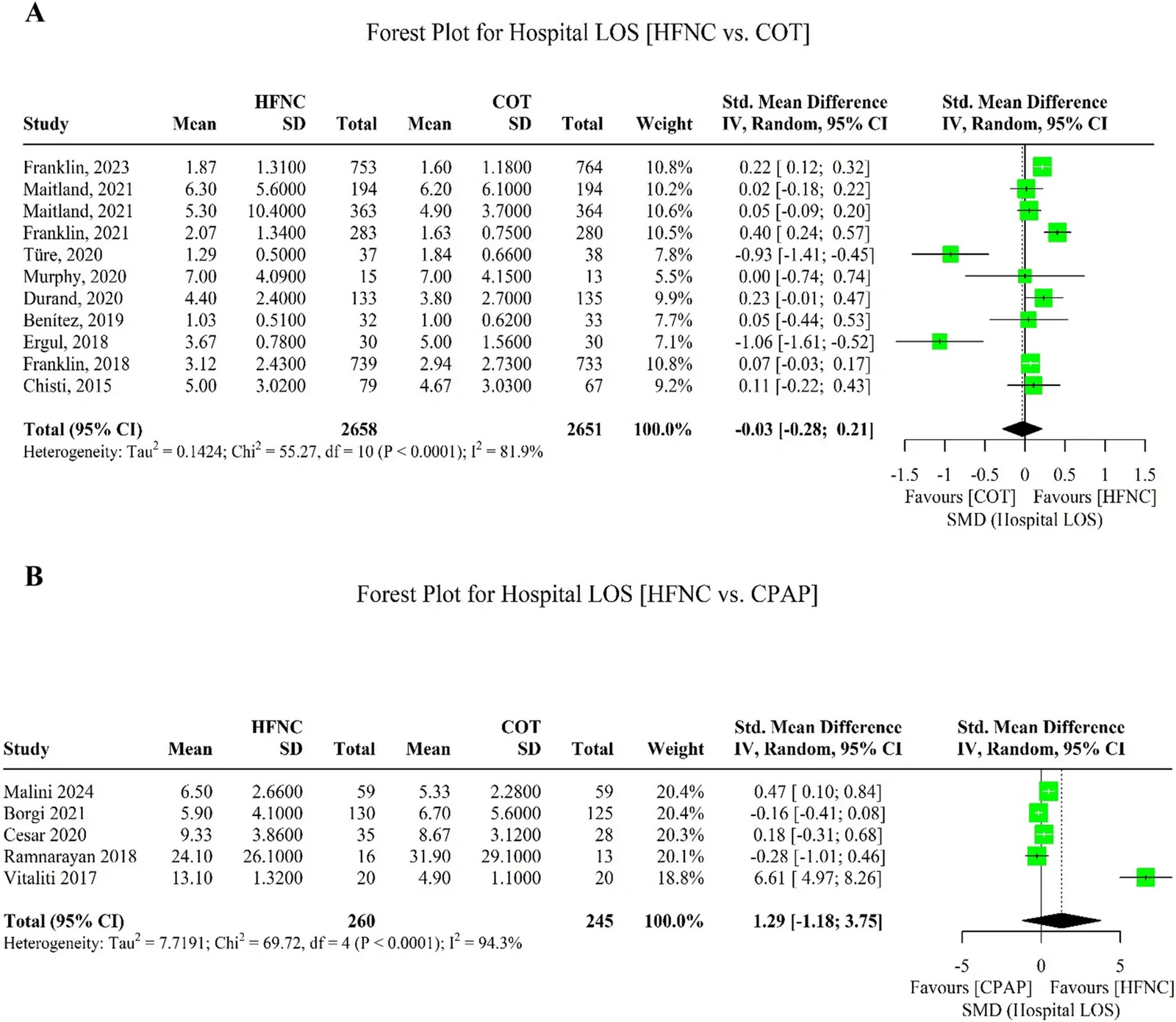
Forest plot for the pairwise comparison of hospital LOS (days); (**A**) high-flow nasal cannula (HFNC) therapy vs. conventional oxygen therapy (COT), and (**B**) high-flow nasal cannula (HFNC) therapy vs. continuous positive airway pressure (CPAP)

Ten trials assessed the impact of HFNC compared to COT on hospital LOS, involving a total of 5,309 children [13, 23, 24, 26, 27, 29, 31, 33, 34, 40]. In addition, five trials compared HFNC to CPAP in 505 children [12, 14, 28, 35, 38]. The findings showed no significant differences in hospital LOS between HFNC and COT (MD: -0.031, 95% CI: -0.28 to 0.21, *P* = 0.823) or between HFNC and CPAP (MD: -1.285, 95% CI: -1.18 to 3.75, *P* = 0.306). Furthermore, there was a high level of heterogeneity among the studies regarding hospital LOS for COT (I²: 81.9%, *P* < 0.001) and for CPAP (I²: 94.3%, *P* < 0.001) (Fig. 7A and B).

#### Hospital mortality

**Fig. 8:**
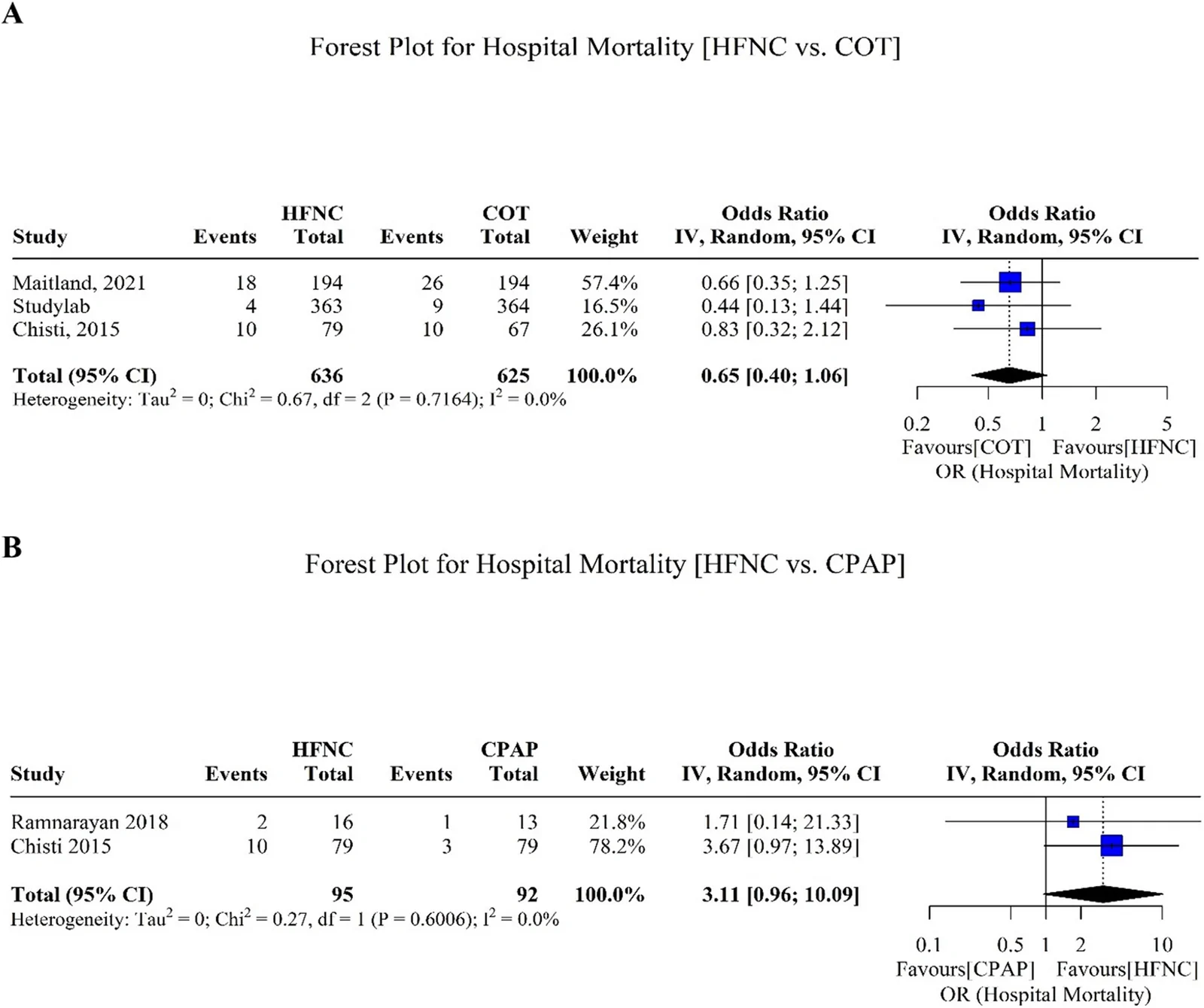
Forest plot for the pairwise comparison of hospital mortality; (**A**) high-flow nasal cannula (HFNC) therapy vs. conventional oxygen therapy (COT), and (**B**) high-flow nasal cannula (HFNC) therapy vs. continuous positive airway pressure (CPAP)

Two trials examined the impact of HFNC compared to COT on hospital mortality rates in a total of 1,261 children [13, 40]. Additionally, two trials investigated HFNC versus CPAP in 187 children [35, 40]. The results showed no significant differences in hospital mortality between HFNC and COT (OR: 0.654, 95% CI: 0.4–1.06, *P* = 0.085) or between HFNC and CPAP (OR: 3.11, 95% CI: 0.96–10.09, *P* = 0.059). Additionally, no significant heterogeneity was observed among the studies regarding hospital mortality rates for COT (I²: 0%, *P* = 0.716) or for CPAP (I²: 0%, *P* = 0.606) (Fig. 8A and B).

### Exploration of heterogeneity

The Galbraith plots were generated to evaluate the presence and degree of heterogeneity among the included studies comparing HFNC with COT (Supplementary file, Figure S1) and HFNC with CPAP (Supplementary file, Figure S2). Overall, most Galbraith plots indicated an absence of significant heterogeneity among the studies. However, the Galbraith plot assessing hospital LOS between the HFNC and COT groups revealed two studies that fell outside the 95% confidence limits, indicating the presence of significant heterogeneity among the included studies for this particular outcome.

### Sensitivity analysis

Sensitivity analyses were performed for both types of studies comparing HFNC with COT (Supplementary file, Figures S3-S5). and CPAP (Supplementary file, Figures S6-S8) for each outcome. In these analyses, each included study was sequentially excluded, and the effect sizes were recalculated. The sensitivity analyses demonstrated that the results remained stable when individual studies were excluded for each outcome in both comparative groups. Overall, the meta-analysis results showed minimal change, confirming the robustness of our findings.

### Publication bias

Publication bias was evaluated using Begg’s test, Egger’s test, and graphical analysis of funnel plots for studies comparing HFNC with COT and CPAP across various outcomes.

For the comparison between HFNC and COT (Supplementary file, Figure S9), the symmetry of the funnel plots revealed no indication of publication bias concerning treatment failure, intubation rate, and hospital mortality. However, a visual assessment of the funnel plots for duration of oxygen therapy, PICU LOS, and hospital LOS suggested the possibility of publication bias. Correspondingly, results from Egger’s and Begg’s tests indicated no significant publication bias for the following outcomes: treatment failure (Egger’s = 0.679, Begg’s = 0.325), intubation rate (Egger’s = 0.309, Begg’s = 0.174), duration of oxygen therapy (Egger’s = 0.175, Begg’s = 0.652), PICU LOS (Egger’s = 0.189, Begg’s = 0.117), hospital LOS (Egger’s = 0.115, Begg’s = 0.312), and hospital mortality (Egger’s = 0.730, Begg’s = 0.602).

In the comparison of HFNC versus CPAP (Supplementary file, Figure S10), visual inspection of the funnel plots indicated a slight degree of publication bias for all outcomes except for hospital mortality. However, Egger’s and Begg’s tests showed no significant publication bias for all outcomes: treatment failure (Egger’s = 0.534, Begg’s = 0.458), intubation rate (Egger’s = 0.155, Begg’s = 0.532), duration of oxygen therapy (Egger’s = 0.645, Begg’s = 0.602), PICU LOS (Egger’s = 0.159, Begg’s = 0.497), hospital LOS (Egger’s = 0.154, Begg’s = 0.327), and hospital mortality (Egger’s = 0.458, Begg’s = 0.705).

### Subgroup analysis

Subgroup analysis was conducted based on the type of respiratory failure in children, specifically focusing on bronchiolitis, pneumonia, and asthma. This analysis was performed only when there were two or more studies available for each type of respiratory failure and its associated outcomes (Supplementary file, Figures S11-S20). It is important to note that some studies only specified children with acute ARF without detailing the underlying cause [23, 24, 35, 37, 38].

The analysis revealed that HFNC therapy significantly reduced the risk of treatment failure compared to COT in children with pneumonia (OR: 0.603, 95% CI: 0.41–0.89, *P* = 0.01), bronchiolitis (OR: 0.453, 95% CI: 0.36–0.58, *P* < 0.001), and respiratory disorders (OR: 0.547, 95% CI: 0.35–0.86, *P* = 0.008). No significant heterogeneity was observed among the studies for pneumonia, respiratory disorders, and bronchiolitis (I²: 0%, *P* > 0.05), based on two [13, 40], two [24, 37] and four studies [29, 33, 34, 39], respectively (Supplementary file, Figure S11).

Additionally, the subgroup analysis indicated a significant increase in hospital LOS in the HFNC group compared to the COT group for children with respiratory disorders (MD: 0.301, 95% CI: 0.12 to 0.48, *P* = 0.028). Again, no significant heterogeneity was detected among these studies (I²: 71.8%, *P* = 0.06), based on two studies [23, 24] (Supplementary file, Figure S15).

## Discussion

The comprehensive and up to date systematic review and meta-analysis conducted on the efficacy and safety of HFNC in comparison to CPAP and COT in treating pediatrics ARF yielded critical insights into the comparative effectiveness of these respiratory support modalities. The analysis demonstrated that HFNC significantly reduced the risk of treatment failure in comparison to COT. However, when juxtaposed with CPAP, HFNC illuminated inconclusive trends with an increased risk of treatment failure, intubation rates, and, notably, an alarming increase in hospital mortality rates. Yet, these results did not attain statistical significance, emphasizing the need for cautious interpretation. Furthermore, no significant differences were observed across all groups concerning the duration of oxygen therapy, PICU LOS, or hospital LOS.

The pooled data indicated that HFNC significantly lowered the risk of treatment failure by 49.1% when compared to COT in pediatric patients with ARF. Furthermore, subgroup analysis revealed that the risk of treatment failure in the HFNC group was reduced by 39.7% in children with pneumonia and by 54.7% in those with bronchiolitis, compared to the COT group. This finding aligns with previous studies which concluded that HFNC provides better physiological outcomes and lower treatment failure rates than COT in pediatric patients with bronchiolitis or respiratory distress [16, 17, 41, 42]. The mechanism behind this effectiveness can be attributed to the ability of HFNC, which delivers heated, humidified oxygen at high flow rates, generating low levels of positive end-expiratory pressure (PEEP) and reducing the work of breathing. These mechanisms improve oxygenation and ventilation, particularly in moderate respiratory distress, by flushing out anatomical dead space and maintaining alveolar recruitment [5, 43–45]. In contrast, COT lacks these benefits, making it less effective in managing moderate to severe pediatrics ARF.

However, when comparing HFNC to CPAP, our results exhibited concerning trends favoring CPAP, specifically showing an increased risk of treatment failure, intubation rates, and, most notably, a near-significant increase in hospital mortality (OR: 3.11, *P* = 0.059). While these findings did not reach strict statistical significance, the mortality signal warrants extreme caution and cannot be ignored. These trends may be explained by CPAP’s greater efficacy in recruiting collapsed alveoli and delivering consistent, higher levels of positive airway pressure compared to the variable and generally lower positive end-expiratory pressure (PEEP) generated by HFNC, which is crucial in treating severe respiratory failure characterized by significant alveolar collapse [44, 45]. Consequently, our findings suggest that HFNC should not be considered a direct substitute for CPAP in severe cases. The concerning mortality signal identified in this analysis underscores the urgent need for future adequately powered, non-inferiority randomized controlled trials comparing HFNC and CPAP in severe pediatric acute respiratory failure, with mortality as a primary endpoint, aligning with recent calls from other systematic reviews and meta-analyses [18, 42, 46, 47].

The lack of statistical significance in our meta-analysis implies that HFNC might still be an appropriate treatment alternative in specific clinical situations, particularly when CPAP is either unavailable or not tolerated by the patient. Additionally, Buendía et al. [18], demonstrated that while CPAP is linked to a lower risk of treatment failure, it is also associated with a higher incidence of adverse events (skin lesions, poor system tolerance, abdominal distension) compared to HFNC. Nonetheless, even though the increased treatment failure rates associated with HFNC compared to CPAP are not statistically significant, they underscore the need for careful evaluation in clinical practice. Several factors could be contributing to the inconclusive trend observed in our results. Variations in study methodologies could lead to inconsistent outcomes; how patients respond to respiratory support may differ based on underlying conditions such as bronchiolitis or pneumonia or asthma; and variability in the definition of treatment failure across different studies may further complicate these inconsistencies. All these elements suggest that while HFNC may have its place in clinical settings, further research is needed to clarify its effectiveness compared to CPAP in various respiratory conditions.

Interestingly, no significant differences were observed between HFNC, COT, and CPAP groups regarding duration of oxygen therapy, PICU LOS or hospital LOS, which are consisting with previous studies [18, 42]. This suggests that these respiratory support modalities may yield similar outcomes in certain clinical scenarios. However, the findings contrast with study by Dafydd et al. [16], reported a significant reduction in oxygen therapy duration and hospital LOS favoring HFNC over COT. These discrepancies may arise from differences in study design, patient populations, disease severity, or institutional protocols. For example, variations in baseline characteristics, such as age, comorbidities, or underlying respiratory conditions, could influence outcomes. Additionally, the significant increase in hospital LOS for HFNC in our subgroup analysis may reflect differences in discharge criteria, clinician practices, or the severity of cases assigned to HFNC. These inconsistencies underscore the impact of contextual and methodological factors on study outcomes. In conclusion, while HFNC, COT, and CPAP appear comparable in some settings, further research is needed to identify specific patient populations and clinical conditions where HFNC may offer distinct advantages, ensuring more targeted and effective use of respiratory support therapies.

The findings underscore the importance of individualized respiratory support strategies in pediatrics ARF. While HFNC is a valuable tool for reducing treatment failure compared to COT, its role relative to CPAP remains less clear, particularly in severe cases. The non-significant increase in mortality risk with HFNC compared to CPAP warrants cautious interpretation and further investigation. Larger, well-designed randomized controlled trials are needed to clarify the comparative effectiveness of HFNC and CPAP in severe pediatrics ARF, particularly in subgroups defined by disease severity and etiology.

### Strengths and limitations

The strengths of this meta-analysis include its significant sample size and the structured approach to data synthesis, comprising a well-defined inclusion criterion and an exhaustive literature search across multiple reputable databases. This comprehensive methodology enhances the robustness of the findings, offering insights that can guide clinical practices in pediatric respiratory care. Nevertheless, the study is not without limitations. The reliance on RCTs, while generally seen as a strength, raises concerns about generalizability; the included trials may not adequately represent the full spectrum of pediatric ARF cases encountered in clinical practice. Furthermore, there may be publication bias; studies with negative results are often less reported, potentially skewing the apparent efficacy of HFNC. Additionally, the substantial variation in study design, population characteristics, and clinical endpoints across trials introduces heterogeneity that complicates direct comparisons of results.

Furthermore, several methodological and statistical limitations must be acknowledged to properly contextualize our findings. First, substantial unexplained statistical heterogeneity (I² values exceeding 80%) was observed in several secondary outcomes, such as the duration of oxygen therapy and length of stay. While our leave-one-out sensitivity analyses confirmed the stability of the effect directions, we acknowledge that this high heterogeneity inherently limits the precision of our pooled effect estimates. This heterogeneity likely stems from clinical and methodological variations across the included trials, such as differing definitions and thresholds for ‘treatment failure,’ varying baseline severities of respiratory failure, and inconsistent device protocols (e.g., varying HFNC flow rates or CPAP interfaces like bubble vs. ventilator CPAP). Second, although formal statistical tests for publication bias (Egger’s and Begg’s) were non-significant, visual inspection of the funnel plots for several outcomes revealed asymmetry, suggesting potential small-study effects or publication bias. This visual asymmetry further tempers confidence in the absolute precision of some pooled estimates. Finally, the inability to blind participants and personnel to the respiratory support modalities resulted in a high risk of performance bias across all included trials, which is an inherent limitation in device-comparison trials.

## Conclusion

This systematic review and meta-analysis elucidate the effectiveness of HFNC as a promising alternative to COT in reducing treatment failure in pediatric acute respiratory failure; however, it does not demonstrate equivalence to CPAP. Notably, a concerning, near-significant trend toward increased hospital mortality with HFNC compared to CPAP warrants extreme caution and necessitates further investigation through adequately powered non-inferiority trials before HFNC can be widely recommended as a substitute for CPAP in severe cases. The findings suggest that while HFNC is a valid non-invasive support modality, its comparative efficacy to CPAP remains ambiguous and warrants further investigation, particularly through well-designed multicenter trials that could provide clarity on its role in pediatric respiratory management. Consequently, clinicians must exercise measured judgment when choosing between HFNC and established therapies, taking into account individualized patient profiles and the complexity of care required. As this field evolves, ongoing research is imperative to refine therapeutic strategies and enhance outcomes for pediatric patients experiencing ARF. Furthermore, as highlighted by recent expert consensus, future multicenter trials must prioritize the standardization of clinical outcome definitions, particularly for ‘treatment failure,’ to minimize methodological heterogeneity and improve the precision of subsequent meta-analyses.

## Supplementary information

Supplementary Material 1.

## Data Availability

A majority of the data analyzed during this study are included in this published article. However, the data that support the findings of this study are available from the corresponding author upon reasonable request.

## Abbreviations

HFNC: High-Flow Nasal Cannula
CPAP: Continuous Positive Airway Pressure
COT: Conventional Oxygen Therapy
ARF: Acute Respiratory Failure
PICU: Pediatric Intensive Care Unit
LOS: Length of Stay
OR: Odds Ratio
CI: Confidence Interval
MD: Mean Difference
RCT: Randomized Controlled Trial
NIV: Non-Invasive Ventilation
BiPAP: Bilevel Positive Airway Pressure
PEEP: Positive End-Expiratory Pressure
PRISMA: Preferred Reporting Items for Systematic Reviews and Meta-Analyses
PECO: Population, Exposure, Comparison, Outcome
PROSPERO: International Prospective Register of Systematic Reviews
