# Supplementary material for: Efficacy and safety of high-flow nasal cannula versus continuous positive airway pressure and conventional oxygen therapies in pediatric acute respiratory failure: a systematic review and meta-analysis
Source: BMC Pediatr. 2026 Jul 1;26:913. doi: 10.1186/s12887-026-07220-8 (PMC13617689; doi:10.1186/s12887-026-07220-8)
Supplement: Supplementary file 1 [file 12887_2026_7220_MOESM1_ESM.docx]

**Table S1. Syntax for Search Strategy**

**A. PubMed**

("High-Flow Nasal Cannula"[Mesh] OR "high-flow nasal cannula"[tiab] OR "high flow nasal cannula"[tiab] OR "HFNC"[tiab] OR "high flow oxygen"[tiab] OR "high flow therapy"[tiab] OR "Continuous Positive Airway Pressure"[Mesh] OR "CPAP"[tiab] OR "nCPAP"[tiab] OR "nasal continuous positive airway pressure"[tiab] OR "Oxygen Inhalation Therapy"[Mesh] OR "conventional oxygen therapy"[tiab] OR "COT"[tiab] OR "standard oxygen therapy"[tiab] OR "nasal cannula"[tiab] OR "oxygen mask"[tiab]) AND ("Child"[Mesh] OR "Infant"[Mesh] OR "Adolescent"[Mesh] OR "pediatric*"[tiab] OR "paediatric*"[tiab] OR "child*"[tiab] OR "infant*"[tiab] OR "children"[tiab] OR "adolescent*"[tiab] OR "young patient*"[tiab]) AND ("Respiratory Distress Syndrome"[Mesh] OR "Respiratory Insufficiency"[Mesh] OR "Bronchiolitis"[Mesh] OR "Pneumonia"[Mesh] OR "Asthma"[Mesh] OR "respiratory failure"[tiab] OR "respiratory distress"[tiab] OR "ARDS"[tiab] OR "PARDS"[tiab] OR "acute respiratory failure"[tiab] OR "acute respiratory distress"[tiab] OR "bronchiolit*"[tiab] OR "pneumonia"[tiab] OR "asthma"[tiab] OR "acute lower respiratory tract infection"[tiab] OR "ALRI"[tiab]) AND ("2026/06/15"[Date - Publication])

**B. Scopus**

TITLE-ABS-KEY ( "high-flow nasal cannula" OR "high flow nasal cannula" OR "HFNC" OR "high flow oxygen" OR "high flow therapy" OR "continuous positive airway pressure" OR "CPAP" OR "nCPAP" OR "nasal continuous positive airway pressure" OR "oxygen inhalation therapy" OR "conventional oxygen therapy" OR "COT" OR "standard oxygen therapy" OR "nasal cannula" OR "oxygen mask" ) AND TITLE-ABS-KEY ( "pediatric*" OR "paediatric*" OR "child*" OR "infant*" OR "children" OR "adolescent*" OR "young patient*" ) AND TITLE-ABS-KEY ( "respiratory distress syndrome" OR "respiratory insufficiency" OR "bronchiolitis" OR "pneumonia" OR "asthma" OR "respiratory failure" OR "respiratory distress" OR "ARDS" OR "PARDS" OR "acute respiratory failure" OR "acute respiratory distress" OR "bronchiolit*" OR "acute lower respiratory tract infection" OR "ALRI" )

**C. Web of Science**

TS=( "high-flow nasal cannula" OR "high flow nasal cannula" OR "HFNC" OR "high flow oxygen" OR "high flow therapy" OR "continuous positive airway pressure" OR "CPAP" OR "nCPAP" OR "nasal continuous positive airway pressure" OR "oxygen inhalation therapy" OR "conventional oxygen therapy" OR "COT" OR "standard oxygen therapy" OR "nasal cannula" OR "oxygen mask" ) AND TS=( "pediatric*" OR "paediatric*" OR "child*" OR "infant*" OR "children" OR "adolescent*" OR "young patient*" ) AND TS=( "respiratory distress syndrome" OR "respiratory insufficiency" OR "bronchiolitis" OR "pneumonia" OR "asthma" OR "respiratory failure" OR "respiratory distress" OR "ARDS" OR "PARDS" OR "acute respiratory failure" OR "acute respiratory distress" OR "bronchiolit*" OR "acute lower respiratory tract infection" OR "ALRI" )

|  |  |
| --- | --- |
|  |  |
|  |  |

Figure S1: Galbraith plots to evaluate heterogeneity among included studies that compare HFNC versus COT for primary and secondary outcomes

|  |  |
| --- | --- |
|  |  |
|  |  |

Figure S2: Galbraith plots to evaluate heterogeneity among included studies that compare HFNC versus CPAP for primary and secondary outcomes

A

B

Figure S3: Sensitivity analysis among included studies for (A) treatment failure, (B) intubation rate between HFNC vs. COT

A

B

Figure S4: Sensitivity analysis among included studies for (A) duration of oxygen therapy, (B) PICU length of stay between HFNC vs. COT

A

B

Figure S5: Sensitivity analysis among included studies for (A) hospital length of stay, (B) hospital mortality between HFNC vs. COT

A

B

Figure S6: Sensitivity analysis among included studies for (A) treatment failure, (B) intubation rate between HFNC vs. CPAP

A

B

Figure S7: Sensitivity analysis among included studies for (A) duration of oxygen therapy, (B) PICU length of stay between HFNC vs. CPAP

A

B

Figure S8: Sensitivity analysis among included studies for (A) hospital length of stay, (B) hospital mortality between HFNC vs. CPAP

|  |  |
| --- | --- |
|  |  |
|  |  |

Figure S9: Funnel plots to publication bias among included studies that compare HFNC versus COT for primary and secondary outcomes

|  |  |
| --- | --- |
|  |  |
|  |  |

Figure S10: Funnel plots to publication bias among included studies that compare HFNC versus CPAP for primary and secondary outcomes

Figure S11: Forest plots of subgroup analysis for treatment failure between HFNC and COT

Figure S12: Forest plots of subgroup analysis for intubation rates between HFNC and COT

Figure S13: Forest plots of subgroup analysis for duration of oxygen therapy between HFNC and COT

Figure S14: Forest plots of subgroup analysis for duration of PICU length of stay between HFNC and COT

Figure S15: Forest plots of subgroup analysis for duration of hospital length of stay between HFNC and COT

Figure S16: Forest plots of subgroup analysis for treatment failure between HFNC and CPAP

Figure S17: Forest plots of subgroup analysis for intubation rate between HFNC and CPAP

Figure S18: Forest plots of subgroup analysis for duration of oxygen therapy between HFNC and CPAP

Figure S19: Forest plots of subgroup analysis for PICU length of stay between HFNC and CPAP

Figure S20: Forest plots of subgroup analysis for hospital length of stay between HFNC and CPAP
